# Supplementary figures and images for: Nanoscale organization of ryanodine receptor distribution and phosphorylation pattern determines the dynamics of calcium sparks
Source: PLoS Comput Biol. 2022 Jun 6;18(6):e1010126. doi: 10.1371/journal.pcbi.1010126 (PMC9203011; doi:10.1371/journal.pcbi.1010126)

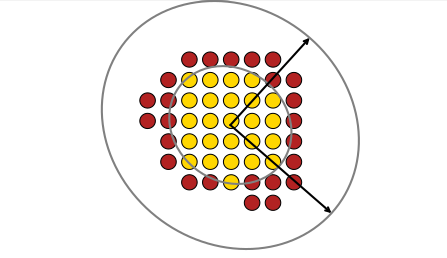

Supplement: S1 Fig — The arrows are vectors showing the eigenvectors of the covariance matrix of the spatial distribution of the RyRs. A first ellipse around the eigenvectors is shown. The ellipse dimensions are decreased (See second, inner ellipse) until the desired number of phosphorylated RyRs is reached (in this case 50%). (TIFF) [file pcbi.1010126.s001.tiff]

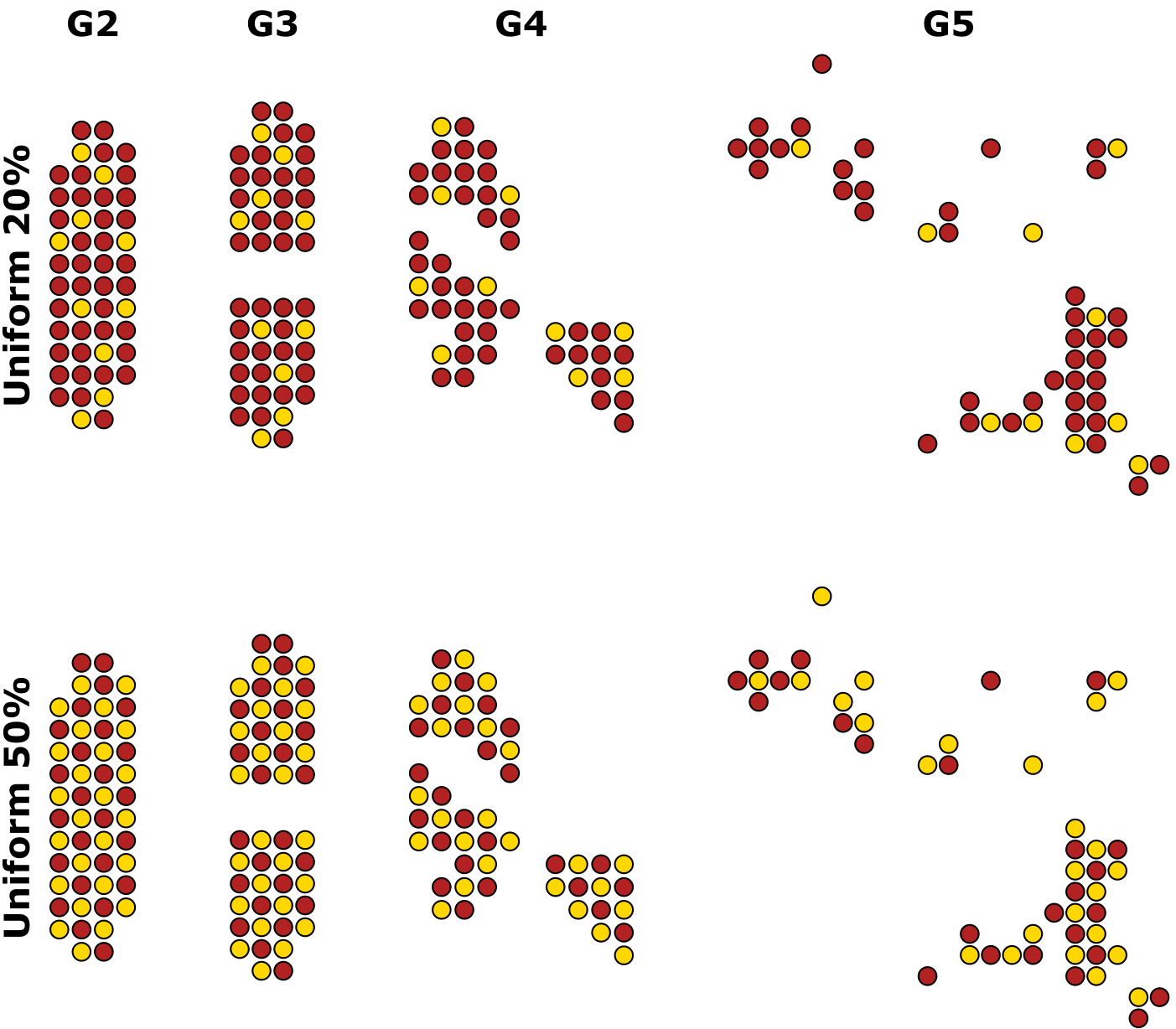

Supplement: S2 Fig — (TIFF) [file pcbi.1010126.s002.tiff]

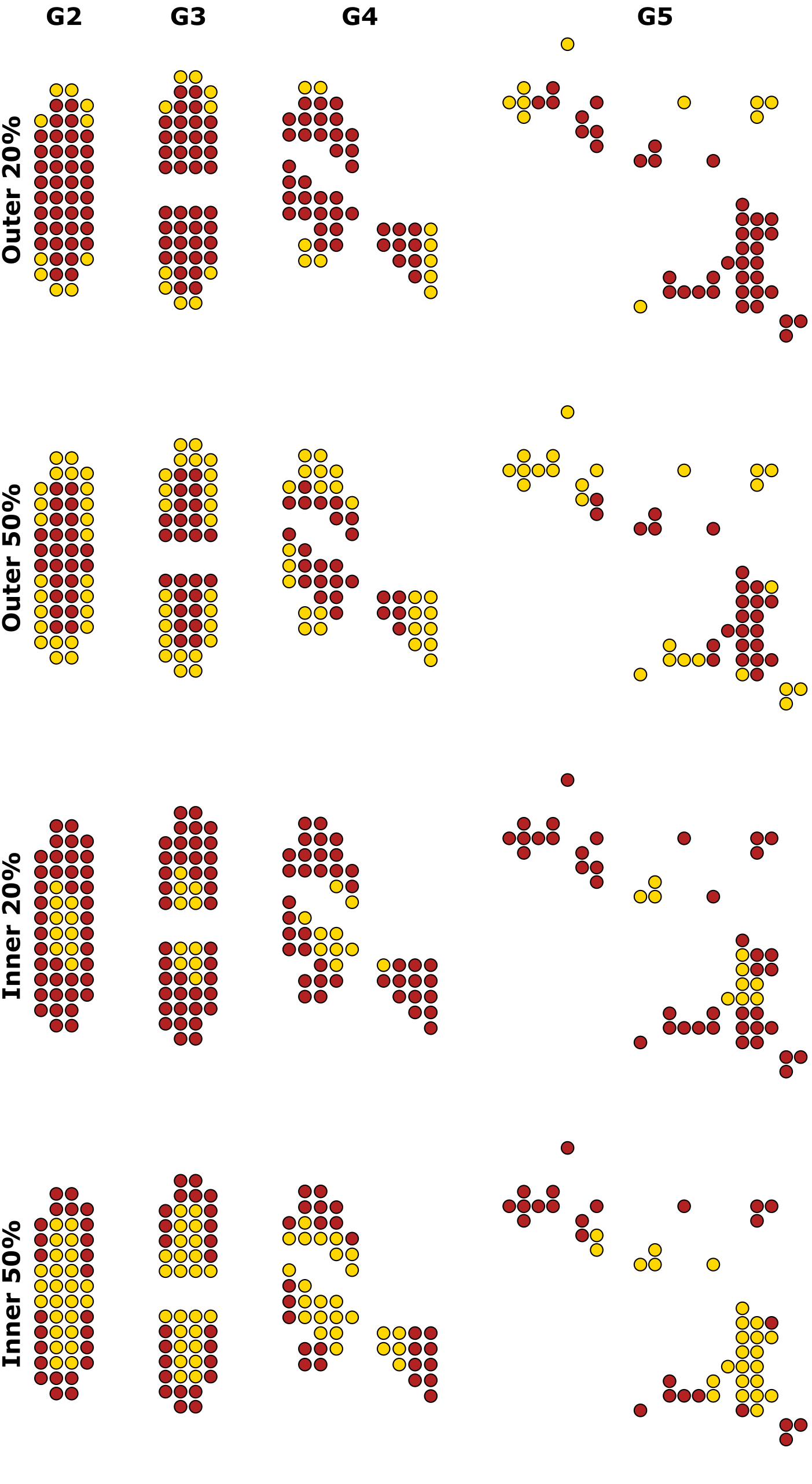

Supplement: S3 Fig — (TIFF) [file pcbi.1010126.s003.tiff]

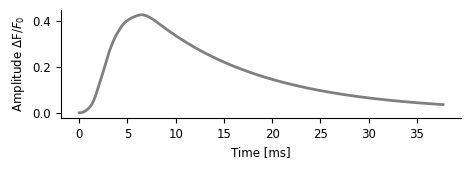

Supplement: S4 Fig — (TIFF) [file pcbi.1010126.s004.tiff]

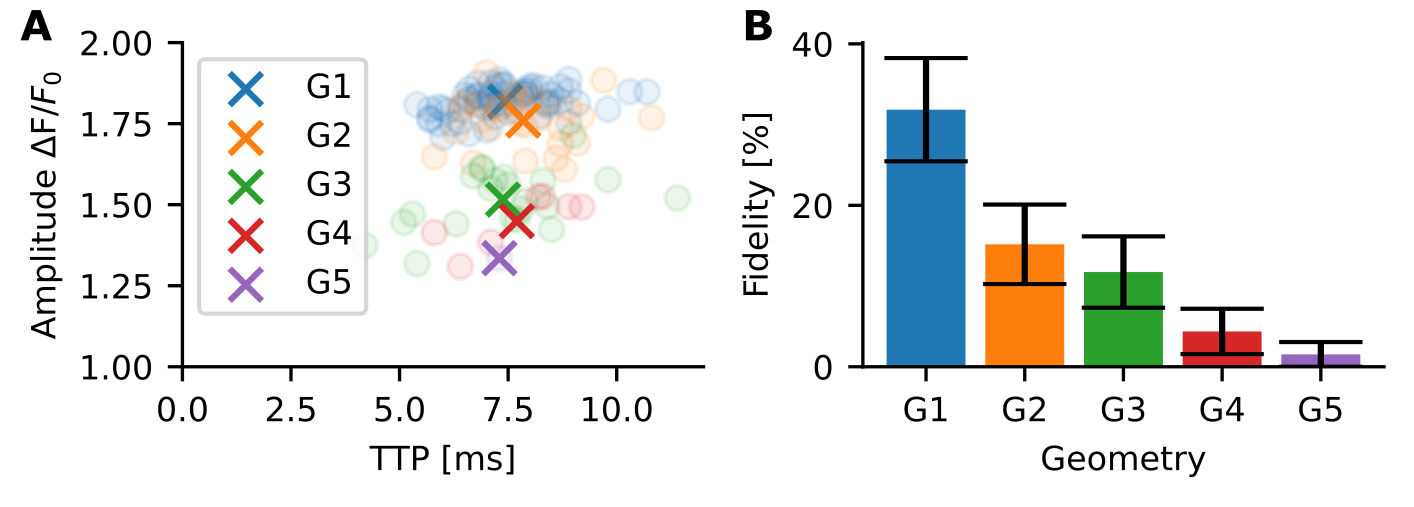

Supplement: S5 Fig — For each geometry 200 simulations were conducted. (A) The distribution created by the relation between TTP and amplitude is shown in a scatter plot shows. The crosses represent the mean values across all simulations. (B) The spark fidelity for each geometry is presented in a bar chart. (TIFF) [file pcbi.1010126.s005.tiff]

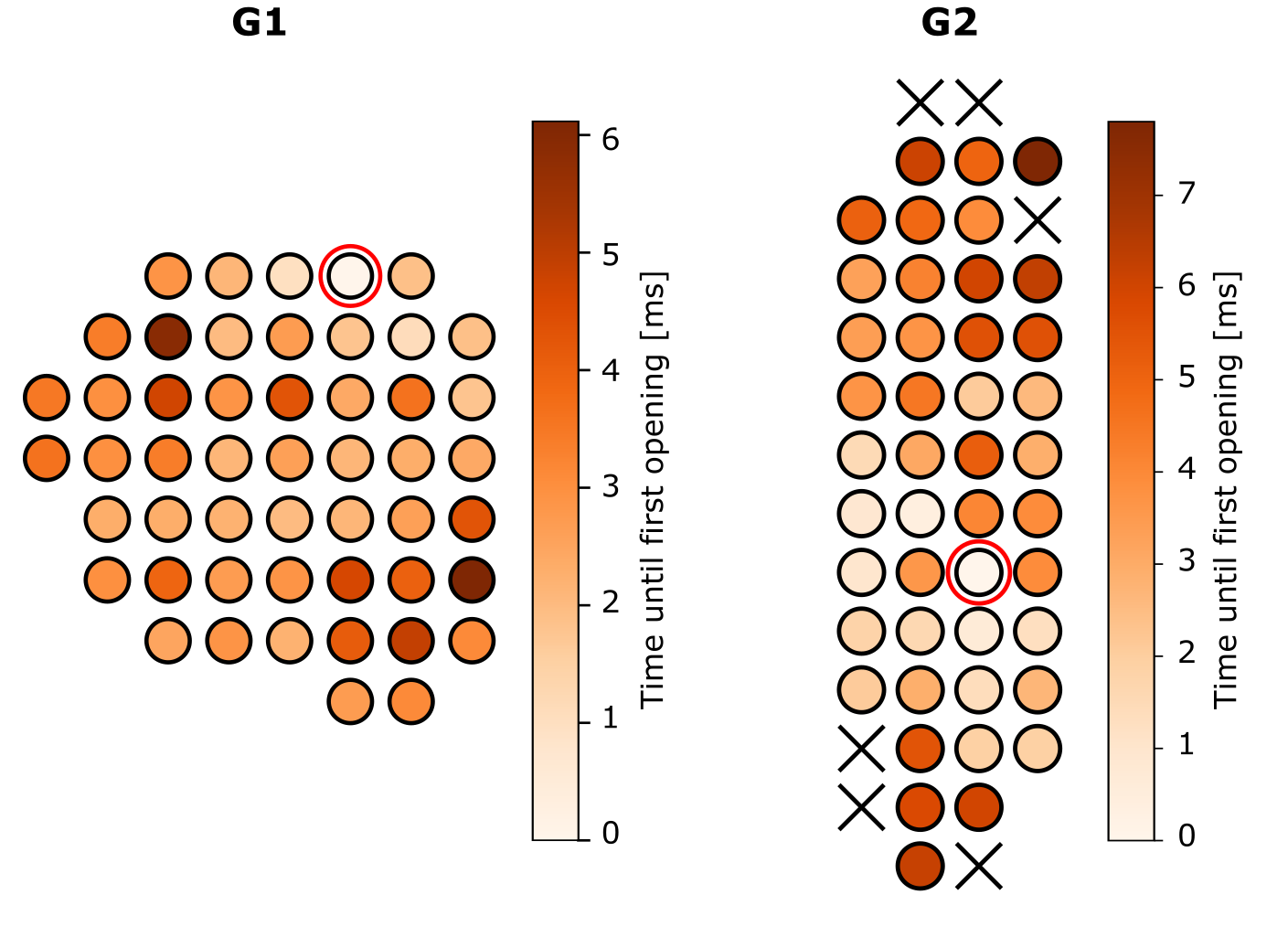

Supplement: S6 Fig — The red circle indicates which RyR was opened to start the simulation. (TIFF) [file pcbi.1010126.s006.tiff]

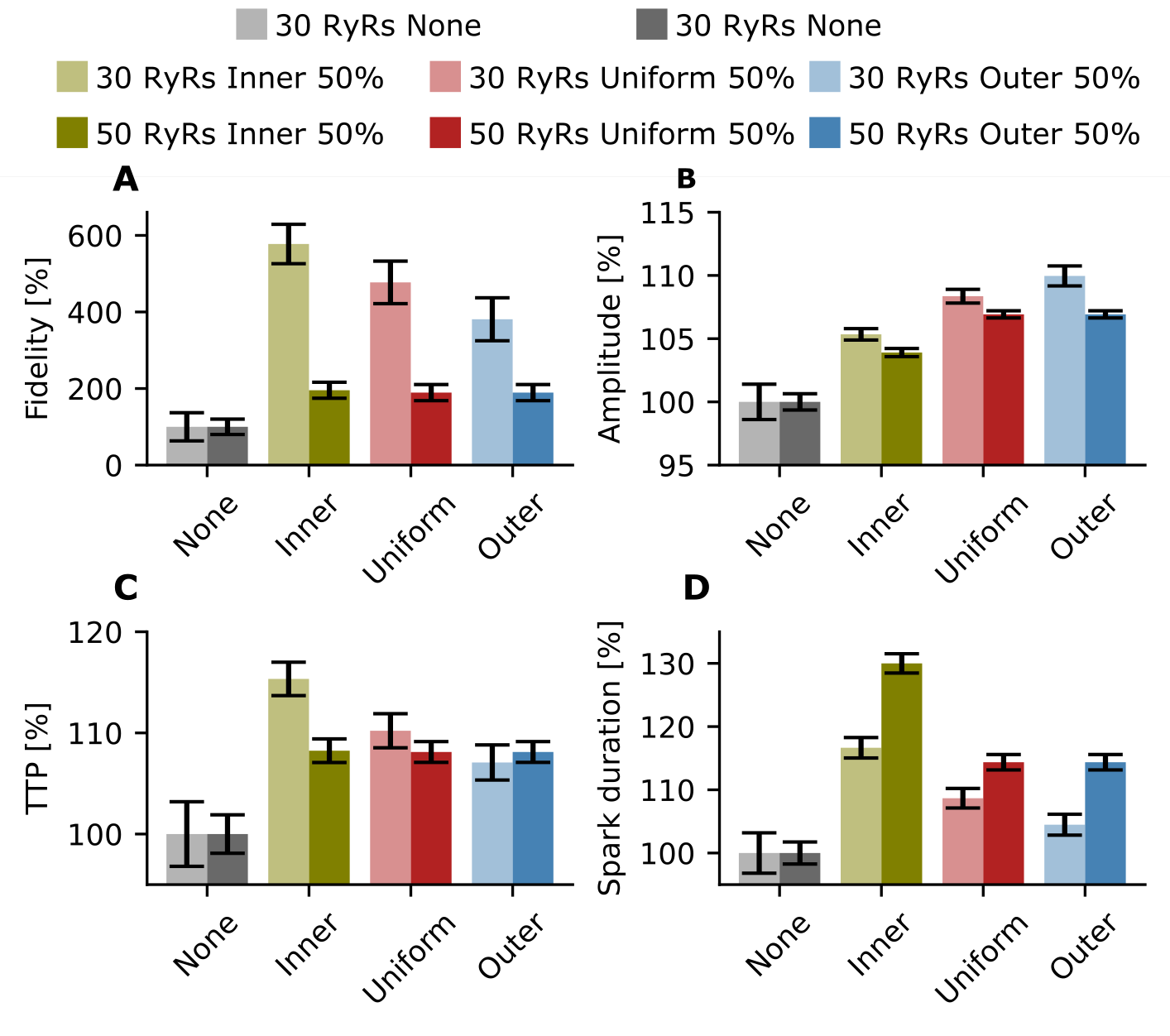

Supplement: S7 Fig — Please note that the bars show relative values; The parameter values for the non phosphorylated case were normalized to 100% and the values shown here represent the relative increase with respect to the non phosphorylated case. (TIFF) [file pcbi.1010126.s007.tiff]

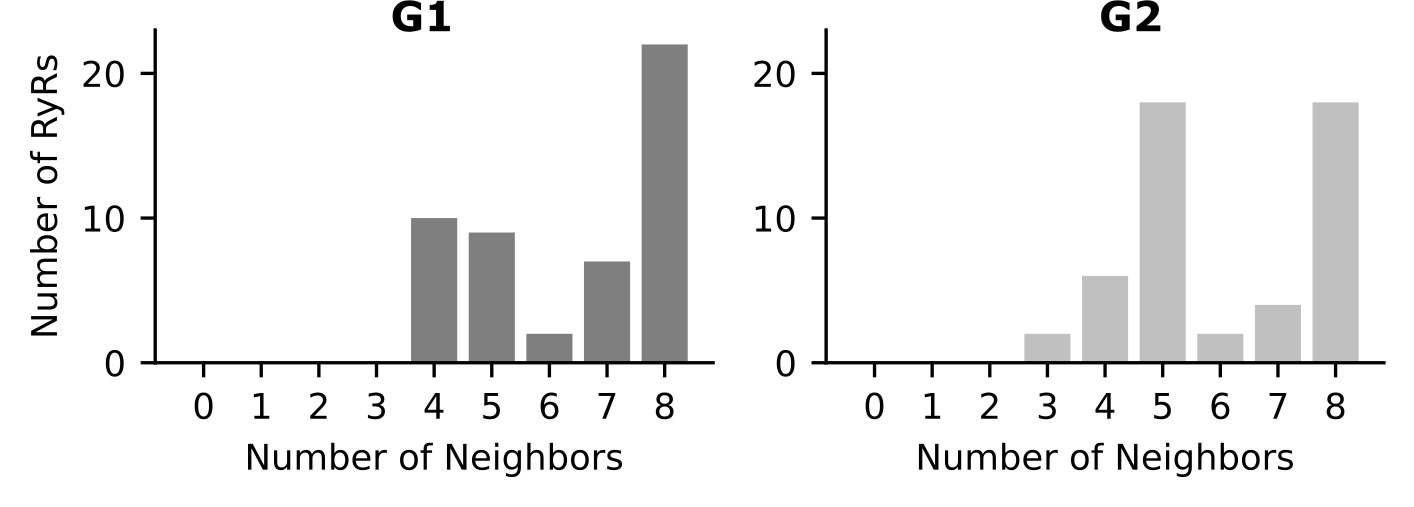

Supplement: S8 Fig — On the left, the histogram for G1 geometry is shown, on the right the histogram for G2 geometry. (TIFF) [file pcbi.1010126.s008.tiff]

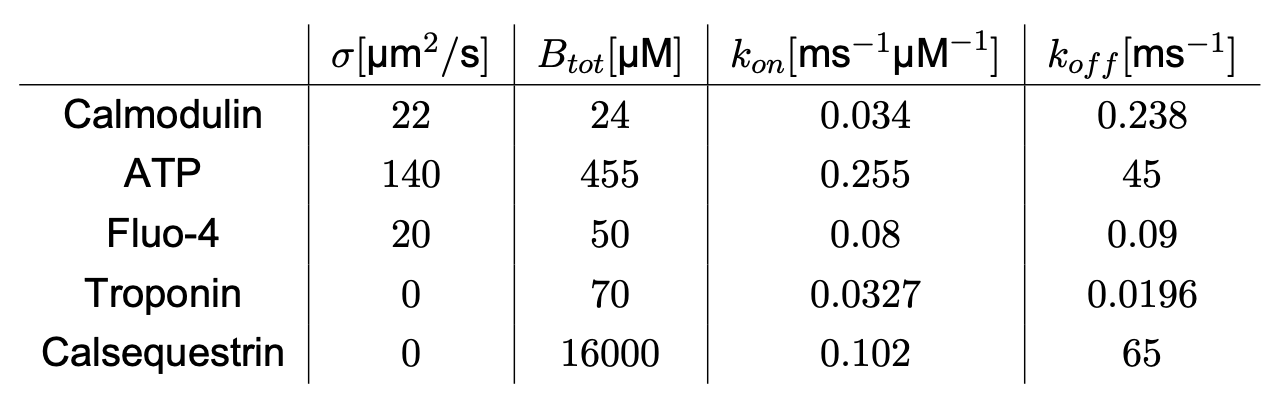

Supplement: S1 Table — (TIFF) [file pcbi.1010126.s009.tiff]

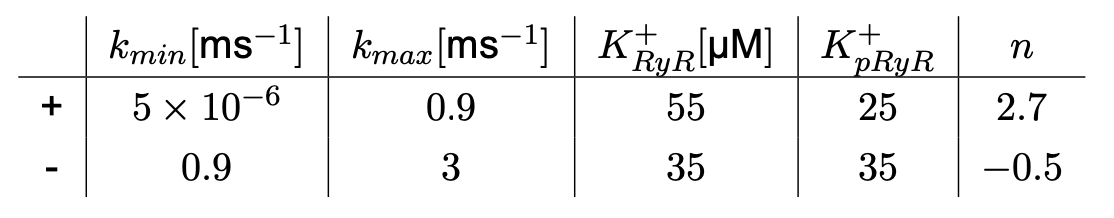

Supplement: S2 Table — (TIFF) [file pcbi.1010126.s010.tiff]

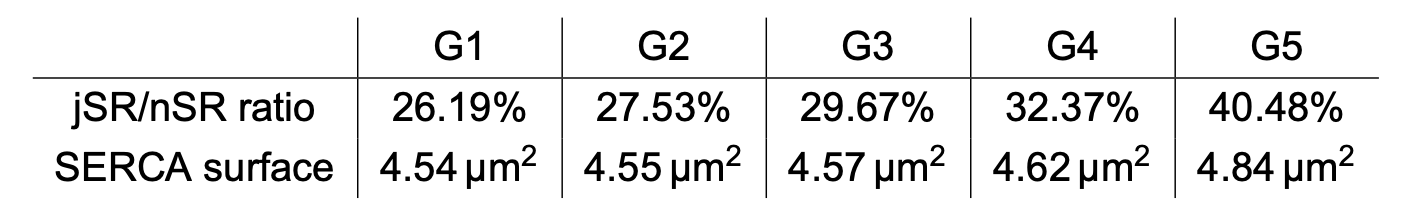

Supplement: S3 Table — (TIFF) [file pcbi.1010126.s011.tiff]
